# Enrichment of Bacteria From Eastern Mediterranean Sea Involved in Lignin Degradation via the Phenylacetyl-CoA Pathway

**DOI:** 10.3389/fmicb.2018.00922

**Published:** 2018-05-09

**Authors:** Hannah L. Woo, Terry C. Hazen

**Affiliations:** ^1^Department of Civil and Environmental Engineering, The University of Tennessee, Knoxville, Knoxville, TN, United States; ^2^Department of Microbiology, The University of Tennessee, Knoxville, Knoxville, TN, United States; ^3^Department of Earth and Planetary Science, The University of Tennessee, Knoxville, Knoxville, TN, United States; ^4^Biosciences Division, Oak Ridge National Laboratory, Oak Ridge, TN, United States

**Keywords:** microbial ecology, lignin, marine biology, degradation, Mediterranean Sea

## Abstract

The degradation of allochthonous terrestrial organic matter, such as recalcitrant lignin and hemicellulose from plants, occurs in the ocean. We hypothesize that bacteria instead of white-rot fungi, the model organisms of aerobic lignin degradation within terrestrial environments, are responsible for lignin degradation in the ocean due to the ocean’s oligotrophy and hypersalinity. Warm oxic seawater from the Eastern Mediterranean Sea was enriched on lignin in laboratory microcosms. Lignin mineralization rates by the lignin-adapted consortia improved after two sequential incubations. Shotgun metagenomic sequencing detected a higher abundance of aromatic compound degradation genes in response to lignin, particularly phenylacetyl-CoA, which may be an effective strategy for marine microbes in fluctuating oxygen concentrations. 16S rRNA gene amplicon sequencing detected a higher abundance of *Gammaproteobacteria* and *Alphaproteobacteria* bacteria such as taxonomic families *Idiomarinaceae*, *Alcanivoraceae*, and *Alteromonadaceae* in response to lignin. Meanwhile, fungal *Ascomycetes* and *Basidiomycetes* remained at very low abundance. Our findings demonstrate the significant potential of bacteria and microbes utilizing the phenylacetyl-CoA pathway to contribute to lignin degradation in the Eastern Mediterranean where environmental conditions are unfavorable for fungi. Exploring the diversity of bacterial lignin degraders may provide important enzymes for lignin conversion in industry. Enzymes may be key in breaking down high molecular weight lignin and enabling industry to use it as a low-cost and sustainable feedstock for biofuels or other higher-value products.

## Introduction

Terrestrial organic carbon cycling in the ocean occurs at significant rates despite most terrestrial organics being thermodynamically stable carbon sources such as cellulose, hemicellulose, and lignin of the plant cell wall (Hedges et al., 1997). About a third of the allochthonous organic carbon in the ocean entering through riverine input is found buried in the sediment (Burdige, 2005) while the rest is putatively degraded within the water column. Of the many factors affecting terrestrial organic carbon utilization like photooxidation (Opsahl and Benner, 1998; Hernes, 2003; Lalonde et al., 2014), biomineralization by microbes is a significant factor (Fichot and Benner, 2014).

Few studies have evaluated lignin degradation in the open ocean compared to more inland locations such as mangroves, ocean margins, or rivers (Dittmar and Lara, 2001; Laurent et al., 2013; Ward et al., 2013; Fichot and Benner, 2014). As the aromatic structure of phenylpropane units in lignin resembles polyaromatic hydrocarbons originating from ocean seeps, marine microbes might catabolize aromatic compounds and possess enzymes such as ring-opening dioxygenases. So far, more isolates in pure culture rather than consortia have been extensively studied for lignin or oil degradation (Arun and Eyini, 2011; Ohta, 2012).

Identifying marine enzymes for lignin degradation would aid the lignocellulosic biofuel industry since one of the major challenges is enzymatically saccharifying the lignocellulosic biomass. The use of ionic liquids (salt-like solvents) in biomass pretreatment requires enzymes to operate in high ionic strength conditions that decrease efficiency (Turner et al., 2003). Marine enzymes will naturally be halotolerant and better retain their conformation in high ionic strength conditions.

Mining enzymes from marine microbes also have several practical advantages in addition to halotolerance. First, less sequencing depth is required to survey marine microbial diversity and species richness compared to soil (Tringe et al., 2005). Second, the ocean’s oligotrophy selects against fastidious fungi and allows bacteria to subsist on lignin. The extreme nutrient limitation of the ocean may trigger the secondary metabolism for microbes that is frequently associated with lignin degradation (Mason et al., 2012). Much less is known about bacterial lignin degradation than fungal despite bacteria being more amenable for commercial enzyme production (Brown and Chang, 2014).

Shotgun metagenomic and 16S rRNA SSU gene sequencing are now common experimental approaches when investigating the microbial diversity of substrate adapted consortia (DeAngelis et al., 2010). Lignin is difficult to quantify and characterize in environmental samples (Hatfield and Fukushima, 2005). A second problem associated with lignin addition experiments followed by cultivation-independent approach is that low levels of other carbon sources may serve as preferred labile carbon source that drastically affect community structure. For the commonly available lignin the most frequent “contaminant” carbon is the hemicellulose polymer xylan and thus control experiments are often performed (DeAngelis et al., 2011).

Here we studied the microbial diversity of xylan- and lignin-adapted Eastern Mediterranean seawater enrichments using sequencing. We hypothesized that microbes in the open ocean must have enzymes capable of degrading lignocellulose. The microbial community used here was originally sampled from the warm surface waters of the Eastern Mediterranean Sea before evolving in a synthetic medium. The Eastern Mediterranean is extremely oligotrophic particularly in phosphate, hypersaline, and well oxygenated even at bottom depths (Techtmann et al., 2015).

## Results

### Mineralization of Lignin and Xylan as Measured by Carbon Dioxide Production

**FIGURE 1:**
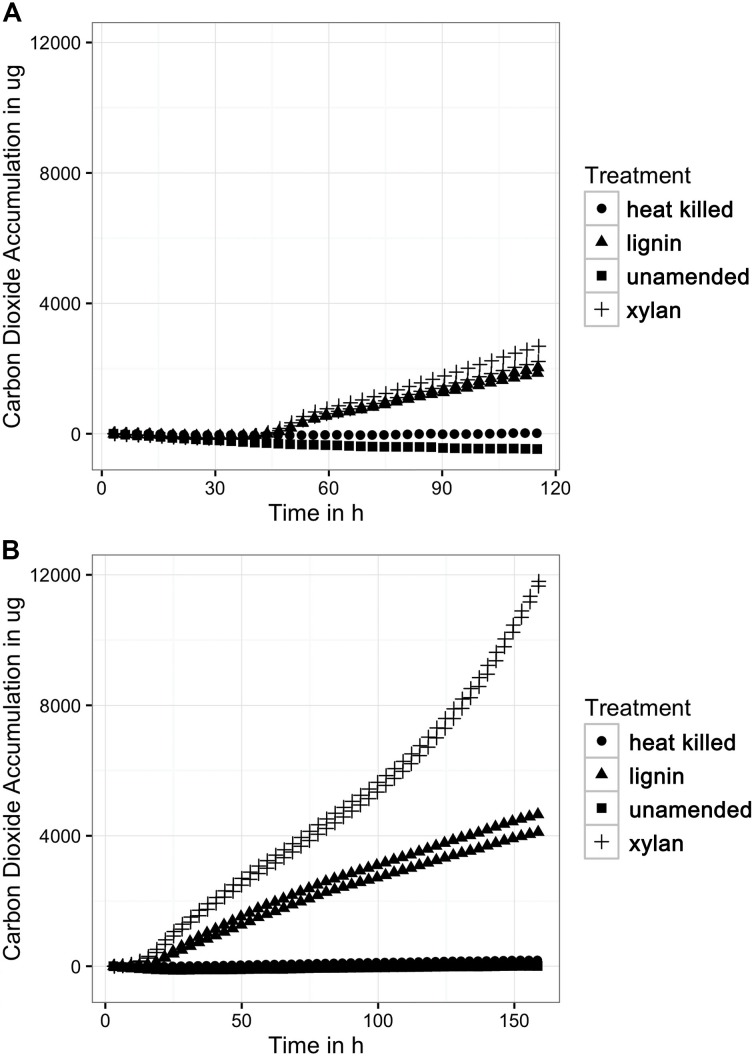
**(A)** Carbon dioxide production from the first incubation of xylan and lignin. The MicroOxyMax respirometer monitored carbon dioxide accumulation in the head space of the three different treatments: the xylan-amended (*n* = 2), lignin-amended (*n* = 2), unamended control (*n* = 1), and abiotic control (*n* = 1). **(B)** Carbon dioxide production from the second xylan and lignin incubation. The first xylan and lignin incubation was transferred to fresh media and substrate for a second incubation, which was also monitored for carbon dioxide accumulation.

The mineralization rates of lignin to CO_2_ by the microbial consortia increased with each transfer to fresh lignin and defined medium (**Figure 1**). The microbial consortia mineralization rates on xylan were higher than on lignin, which suggests that xylan is a more labile carbon source to the marine microbes. It is unlikely that carbon dioxide was produced from carbonates in the media because pH was stable at 8.0 before and after the incubation. Other experimental controls such as an unamended control and abiotic control did not produce significant amounts of carbon dioxide. As expected of oxic cultures, a significant amount of oxygen was consumed by the substrate-adapted consortia. The oxygen uptake (Supplementary Figure S1) strongly correlated linearly with the carbon dioxide accumulation.

The xylan-adapted consortia and the lignin-adapted consortia produced similar amounts of carbon dioxide during the first incubation of approximately 1 week. Both types of consortia produced about 2500 μg of carbon dioxide within the first 120 h (**Figure 1A**) after a lag phase of 53 h of very low respiration. The next incubation took place immediately after the first incubation. A 1.6% inoculum of the first substrate-amended consortium was used to inoculate fresh substrate in fresh artificial seawater media. The second xylan-adapted consortia produced 12,000 μg of carbon dioxide within 150 h, which is nearly five times more carbon dioxide than the first consortia (**Figure 1B**). The second lignin-adapted consortia also improved in mineralization rate but not as much as the xylan-adapted consortia. The lignin enrichment produced about 4000 μg, which was nearly twice as much as the first incubation. In contrast to the first incubation, the second incubation of both substrates did not have a lag phase.

### Shifts in Functional Gene Diversity

**FIGURE 2:**
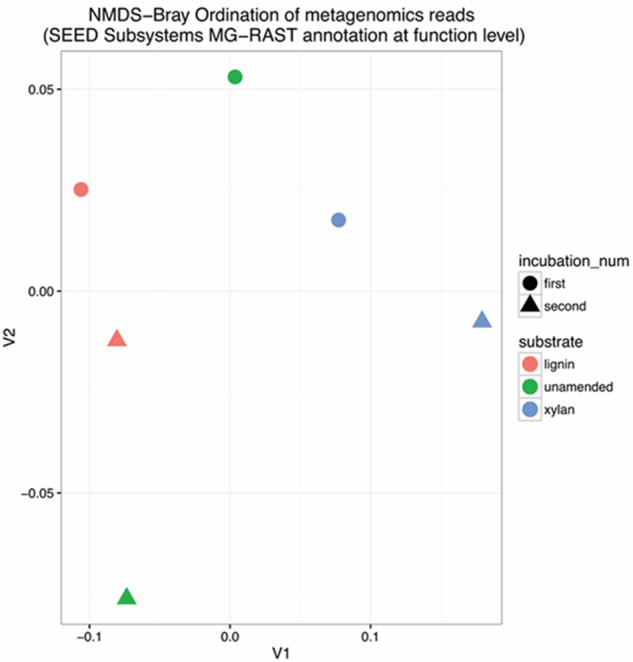
Non-metric Multidimensional Scaling (NMDS) Bray Ordination of functional gene structure. Functional genes were characterized by shotgun DNA sequencing and compared using Bray–Curtis distance before NMDS. Stress was 3.25 × 10^-14^. The substrate is indicated by the color in the ordination plot: red for lignin, green for unamended, blue for xylan. The incubation is indicated by the shape of the point: circle for the first incubation and triangle for the second incubation.

The functional gene diversity of the enrichments was characterized by shotgun metagenomes Illumina MiSeq DNA sequencing. Total shotgun sequences ranged from 0.5 to 7.9 million sequences per sample and had 53–57% average GC content (Supplementary Table S1). The unassembled reads were annotated by the automated MG-RAST pipeline (Meyer et al., 2008) (Supplementary Table S1) using the SEED Subsystems hierarchical organization. The samples were compared using a Non-metric Multidimensional Scaling (NMDS) of Bray–Curtis distances (**Figure 2**) of the number of reads classified at the finest level of functional annotation consisting of 4663 different functions. The first and second incubations of the lignin were more similar to each other than the incubations with xylan. The two incubations of the unamended control were very different.

**FIGURE 3:**
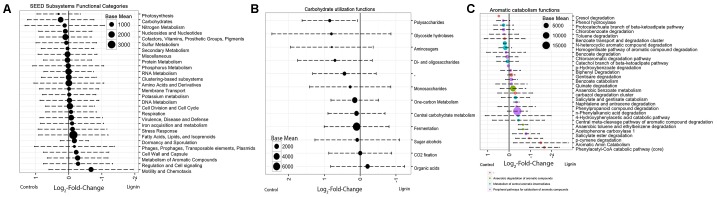
Average log_2_-fold change of differentially abundant functional categories between the two lignin and two xylan microcosms. The log-fold change and base mean were determined by R package, *DESeq2*. Base mean, as represented by the size of the point, is the average of the normalized counts over all samples. Categories are based on SEED Subsystems annotation. Positive log_2_-fold change indicates functions in higher abundance in lignin-amended microcosms. Negative log_2_-fold change indicates functions in higher abundance in xylan-amended microcosms. Error bars represent the standard deviation. **(A)** All functional categories, **(B)** only carbohydrate-related functions, and **(C)** only aromatic catabolism-related functions.

The differential abundance of functional genes between the two xylan-amended consortia and the two lignin-amended consortia was analyzed using *DESeq2*. Positive log_2_-fold changes indicated a greater abundance in the lignin-amended microcosms while negative values indicated a greater abundance in xylan-amended microcosms. Calculated log_2_-fold changes ranged from -0.6743 to +0.3077. The functions associated with carbohydrates and aromatic compound catabolism were the most differentially abundant categories (**Figure 3A**). The abundance of carbohydrate metabolism functions was higher in the xylan-amended microcosms. The average log_2_-fold changes of the 1057 carbohydrate functional genes (Supplementary Figure S2) were negative at -0.245. Meanwhile, the lignin-amended microcosms had more aromatic catabolism genes. 237 genes of the aromatic compound metabolism category were on average +0.268.

Nearly all subcategories of genes related to carbohydrate utilization were more abundant in the xylan-adapted consortia (**Figure 3B**). Among the subcategories of genes in identifiable groups of functions related to carbohydrate utilization, polysaccharides utilization, glycoside hydrolases, di- and oligosaccharides, and monosaccharide utilization were the most differentially abundant groupings. The only functional categories that were more abundant in the lignin-adapted consortia were the organic acids.

For the aromatic metabolism genes, functions that were more abundant in the lignin-adapted consortia were linked to phenylacetyl-CoA catabolism pathway (**Figure 3C**). Phenylpropanoid compound degradation was also more abundant in the lignin-adapted consortia, which included genes for vanillate and ferulate metabolism, two potential breakdown products from lignin. The most differentially abundant functional gene was 3,4-dihydroxyphenylacetate 2,3-dioxygenase (EC 1.13.11.15), which is essential for aromatic compound catabolism. Also, catechol 1,2-dioxygenase was more abundant in lignin-adapted consortia.

The phenylacetyl-CoA-annotated reads within the lignin metagenomes were associated with *Pseudomonas* and *Marinomonas* species. The two lignin enrichment metagenomes had 10,390 annotated reads that fell into the phenylacetyl-CoA core SEED Subsystems category as a closest representative hit. The nearest source organism of each annotated read was determined by using the GenBank record’s source organism. Among the phylogeny associated with the reads matching phenylacetyl-CoA-related genes, *Pseudomonas* species such as *P. fluorescens* str. Pf-5, *P. entomophila* str. L48, various *P. putida* strains, and *Marinomonas* sp. str. MWYL1 were among the five most represented organisms.

### Changes in Microbial Community Composition

The microbial community composition of the lignin-adapted, xylan-adapted, and unamended consortia was characterized using shotgun metagenomics and 16S rRNA gene amplicon sequencing. DNA from each of the two replicates for each treatment and control at each time point was pooled before Illumina MiSeq sequencing. While the shotgun metagenomic reads provided information about all microbial phyla including fungi, bacteria, and archaea, in contrast. 16S rRNA gene amplicon sequencing relied on primers that only target bacteria and archaea but provided better precision than the shotgun approach. Shotgun metagenomics reads were annotated by the MG-RAST pipeline using the M5NR database. 16S rRNA gene amplicon reads were processed into operational taxonomic units (OTUs) and identified by comparison to the Greengenes database (DeSantis et al., 2006) using QIIME UCLUST method (Caporaso et al., 2010). Libraries were normalized by rarefying.

**FIGURE 4:**
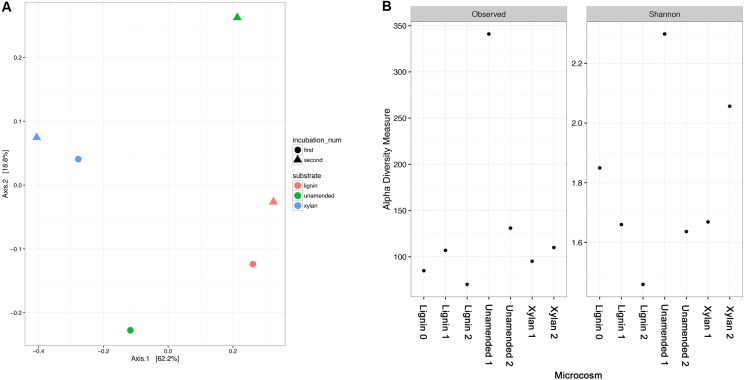
**(A)** Principal coordinate analysis (PCoA) of the microbial community structures from unamended, xylan, and lignin amended microcosms. The microbial community structures of the microcosms were characterized by 16S rRNA gene amplicon sequencing. Microbial communities were compared using a weighted Unifrac distance before PCoA. The first two axes shown together describe about 80% of the variance between the samples. The incubation is indicated by the color of the point in the ordination plot: pink for the earlier incubation, turquoise from the latter. The amended substrate is indicated by the shape of the point: circle for lignin, square for xylan, and triangle for unamended controls. **(B)** Alpha diversity of the microbial community structure from xylan and lignin microcosms using number of OTUs and Shannon diversity index. All indices were calculated using the *phyloseq* package in R.

The community structure of the samples was compared by principal coordinate analysis (PCoA) using weighted Unifrac distances (**Figure 4A**). The lignin- and xylan-adapted consortia were distinct communities. The two incubations of each substrate were similar in community while the two incubations of the control were different. The controls were likely very different between the incubations in part because of differences in richness and that difference was significantly different from one of the controls (**Figure 4B**). The substrate-amended consortia had less observed OTUs than the controls. The control at the first timepoint had the highest number of distinct OTUs, nearly 350, while the second timepoint had less than half than the first. The Shannon diversity index which considers evenness in addition to richness suggested a decreasing trend for lignin-adapted consortia but an increasing trend for xylan-adapted consortia over time. Together these results suggested that a subset of species became dominant in the lignin-adapted consortia. Meanwhile, species became more evenly distributed within the xylan-adapted consortia.

**FIGURE 5:**
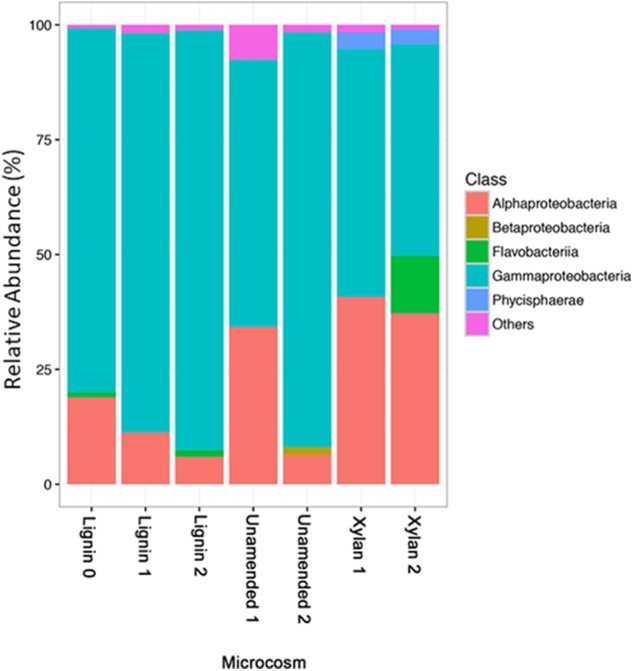
The relative abundances of taxonomic classes identified by 16S rRNA gene amplicon sequencing. Classes that are less than 1% relative abundances are aggregated as others.

The microbial communities of the unamended microcosms and xylan-adapted consortia were compared on three different levels of taxonomy: phylum, class, and order. Rare OTUs with less than 1% of reads were excluded from visualization. At the phylum level, we observed more *Bacteroidetes*, *Planctomycetes*, *Acidobacteria*, and *Actinobacteria* in the unamended and xylan controls (Supplementary Figure S3). Meanwhile, the lignin-adapted consortia consisted mostly of *Proteobacteria* and some *Bacteroidetes*. Of the most abundant classes (**Figure 5**), *Gammaproteobacteria* increased in the lignin microcosms but decreased in the xylan microcosms. Of the *Gammaproteobacteria*, *Oceanospiralles* was the most dominant taxonomic family but found ubiquitously in all treatments (Supplementary Figure S3). *Alphaproteobacteria*, particularly the order *Kiloniellales*, increased in the xylan-amended microcosms (Supplementary Figure S3). *Flavobacteria* is important in xylan microcosms after the last incubation, particularly the genus *Salegentibacter* (Supplementary Figure S3).

Archaea were only present in the first unamended control (Supplementary Figure S3). The orders *Nitrososphaerales* and *Cenarchaeales* of the phylum *Thaumarchaeota* were the most abundant archaeal taxa in the first unamended control (Techtmann et al., 2015). Archaea were less than 0.1% in lignin-adapted and xylan-adapted consortia. The decrease in Archaea abundance corroborates other studies in the Eastern Mediterranean where Archaea became rare upon carbon amendment (manuscript in preparation).

Fungi, like Archaea, were very low in abundance in the samples. Fungi were characterized using shotgun metagenomic unassembled reads. The fungal reads totaled less than 0.04% of any of the substrate-adapted or control metagenomes (Supplementary Table S2). Ascomycetes were 0.04% or less; Basidiomycetes were 0.01% or less. In contrast, Proteobacteria, Bacteroidetes, Actinobacteria, and Firmicutes constituted the majority at 97.98% of shotgun metagenomic reads (Supplementary Table S2). The most abundant Ascomycetes and Basidiomycetes were Nectricia, Trichocomaceae, Debarymycetaceae, Ustilaginaceae, Schizosaccharamycetaceae, and Sordariaceae families (Supplementary Figure S4).

### Potential Lignin-Degrading Species in Lignin-Adapted Consortia

Differential abundance analysis was used to determine which taxa increased with lignin and xylan amendment. This analysis was conducted using the R package *DESeq2* (Love et al., 2014). Rare phyla with less than 0.005% relative abundance were excluded from further analysis. The shotgun metagenome captured a broad diversity of organisms including bacteria, fungi, archaea, plants, and other larger eukaryotes. Invertebrates and vertebrate phyla were excluded from the analysis.

**FIGURE 6:**
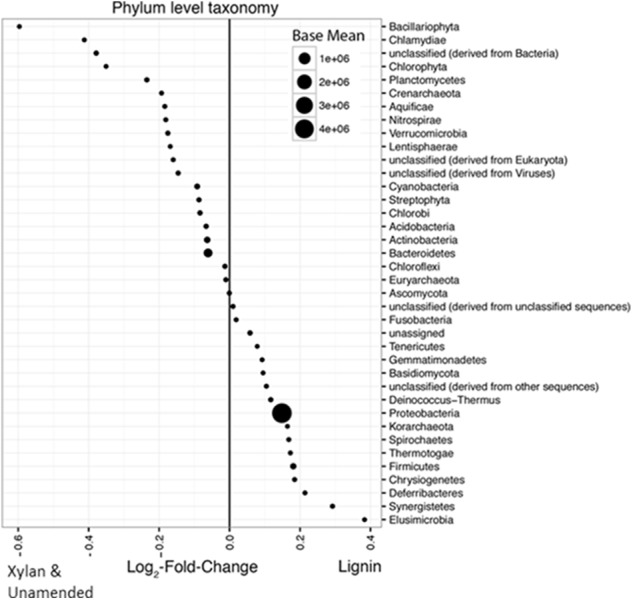
Average log_2_-fold changes of phyla between lignin-adapted consortia and the controls (xylan-adapted and unamended consortia). Abundances from the MG-RAST annotation were compared between the two lignin-adapted consortia and the four non-lignin controls. The first and second incubations were combined for each substrate. The non-lignin controls included both the unamended control and the xylan-adapted consortia. The size of the point indicates its normalized relative abundance. A positive log_2_-fold change indicates higher abundance in lignin-adapted consortia while a negative log_2_-fold change indicates higher abundance in the controls. Base mean is the average of the normalized counts over all samples. Phyla that were less than 0.005% were excluded.

More bacterial phyla than fungal phyla increased in response to lignin. The differences between the lignin-adapted consortia and the controls were small, only up to 0.6 log_2_-fold change different. Phyla Elusimicrobia, Synergistetes, Deferribacteres, Chrysiogenetes, Firmicutes, Thermotogae, Spirochaetes, Korarchaeota, Proteobacteria, Deinococcus-Thermus, Basidiomycota, Gemmatimonadetes, Tenericutes, Fusobacteria, and various unclassifiable groups were found in higher abundance in the lignin-adapted consortia compared to xylan-adapted consortia or the unamended control (**Figure 6**). Two fungal phyla with white rot fungi, *Ascomycota* and *Basidiomycota*, were very low in relative abundance and were in more similar abundances between the controls and lignin-adapted consortia than Bacteria. *Ascomycota* was found in slightly higher abundance in xylan and unamended controls, while *Basidiomycota* was slightly higher in the lignin-adapted consortia.

**FIGURE 7:**
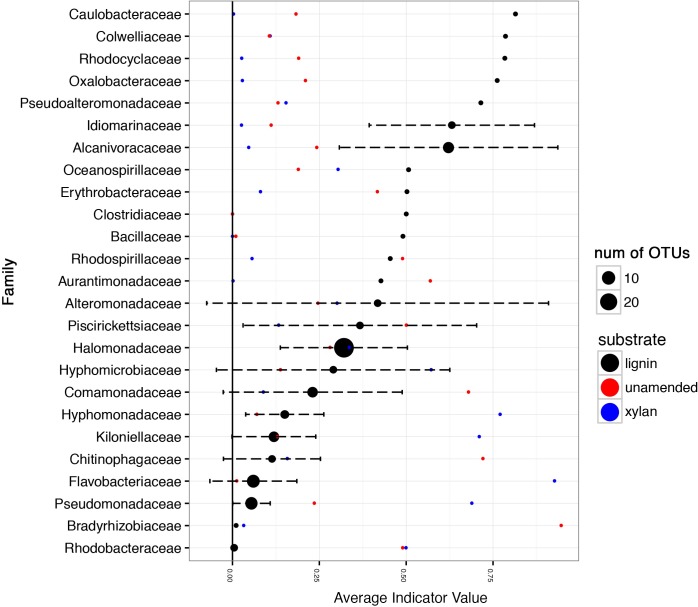
Average indicator values of taxonomic families for lignin amendment. The abundances of the OTUs in the 16S rRNA gene amplicon data were used to calculate the indicator value using the R package labdsv indval function. The first and second incubations were combined for each substrate. The average of the indicator value for lignin is shown by the black points. The error bars indicate the standard deviation of the indicator values. The size of the point indicates how many distinct OTUs are represented in the dataset. Taxonomic families with indicator values less than 0.1 for lignin were excluded from the visualization. The red and blue points indicate indicator value for the unamended control and xylan amendment, respectively.

Indicator value of the taxa detected in the 16S rRNA gene amplicon data considers the fidelity and relative abundance of a taxa for lignin instead of log-fold changes. *Caulobacteraceae*, *Colwelliaceae*, *Rhodocyclaceae*, *Oxalobacteraceae*, *Pseudoaltermonadaceae*, *Idiomarinaceae*, *Alcanivoraceae*, *Oceanospirallaceae*, *Erythrobacteraceae*, and *Clostridicaeae* had indicator values greater than 0.5, with 1.0 being the maximum possible value (**Figure 7**). In contrast, some families such as *Alteromonadaceae*, *Piscirickettsiaceae*, *Halomonadaceae*, and *Hyphomicrobiaceae* were ubiquitous in all consortia regardless of carbon source.

## Discussion

Our objective was to discover the diversity of marine microbes from the Eastern Mediterranean Sea that are capable of degrading lignin and thereby potentially contributing to allochthonous terrestrial organic matter cycling. We hypothesized that oligotrophic and hypersaline marine microbial communities can provide insight into the untapped diversity of bacteria with efficient lignin-degrading enzymes. Evidence of increasing mineralization rates on lignin, changes in microbial community structure, and increases in abundance of aromatic catabolism gene abundance support the notion of the Eastern Mediterranean seawater possessing high potential for lignin degradation.

The lignin mineralization rates by the Eastern Mediterranean microbial consortia improved with transfers due to changes in the microbial community. The mineralization rates in the lignin-amended consortia nearly doubled between the first and second incubation. The mineralization rate at the end of the second incubation had not leveled off, indicating the potential to produce more carbon dioxide with more time. Abiotic processes are unlikely to be responsible for the increase in mineralization rates since the lignin and the defined media were fresh at each transfer and the incubation conditions remained constant. The results of the shotgun metagenomics and 16S rRNA gene amplicon sequencing analyses suggested that the microbial community structure became less diverse between transfers on lignin as expected of substrate-adapted consortia over time (DeAngelis et al., 2010).

The increase in mineralization within the lignin-amended microcosms coincided with shifts in bacterial rather than fungal populations. *Gammaproteobacteria* was the most abundant class of microorganism overall in these microcosms. Similar observations of higher efficiency by bacteria like *Gammaproteobacteria* than fungi for lignocellulose degradation has been found before in marine sediments (Gontikaki et al., 2015). Multiple taxonomic families of *Gammaproteobacteria* bacteria, such as the mesophilic and halophilic *Idiomarinaceae*, increased in abundance in the presence of lignin compared to the controls. The *Alphaproteobacteria* family, *Caulobacteraceae*, was an indicator species for lignin and has been associated with lignin before in tropical forest soils, another oligotrophic environment where nutrients limit fungal growth (DeAngelis et al., 2011). Other indicator species for lignin are known marine oil-degrading species such as *Colwelliaceae* (Mason et al., 2014) and *Oceanospirillaceae* that can quickly respond when carbon becomes available in excess like during an oil spill (Mason et al., 2012). *Alteromonadaceae* was found in the Eastern Mediterranean Sea to respond and proliferate rapidly to oil amendments (Liu et al., 2017) and was also enriched in our lignin-amended treatments. The ability of oil-degrading microbes to degrade other recalcitrant aromatic compounds like lignin is not surprising.

Meanwhile, fungi remained low and stable in abundance in all consortia from the Eastern Mediterranean. Among the few fungal taxa that were identified, *Basidiomycetes* slightly increased in the presence of lignin, while *Ascomycetes* slightly decreased. Relative abundance of *Ascomycetes* was slightly higher than *Basidiomycetes* in general. Fungi have been shown to be rare and low in diversity by another cultivation-independent study (Bass et al., 2007). Few marine white-rot basidiomycetes have been described in the literature, with much of the knowledge about lignocellulose marine fungi being from mangroves (Pointing and Hyde, 2000). A previous study compared bacterial and fungal impact on lignocellulose degradation in salt marsh sediment and also showed bacteria were dominant degraders of detritus (Benner et al., 1984). Despite the changes in the community in response to lignin, the possibility remains that low abundance microbes are influencing the increase in mineralization as this is common.

Despite the improvements in mineralization rate, the Eastern Mediterranean consortia likely do not degrade lignin as quickly as white-rot fungi in pure culture. Under specific optimized conditions a soil basidiomycete white-rot fungus, *Phanerochaete chrysosporium*, has been shown to mineralize 1% of a radiolabeled synthetic lignin into carbon dioxide every 24 h (Boyle et al., 1992). However, *P. chrysosporium* is an exceptional case as other white-rot fungal strains from marine environments have also been tested with radiolabeled lignin and had much lower mineralization rates, less than 5% in 60 days (Sutherland et al., 1982).

Based on the functional gene structure of the lignin-adapted consortia, aerobic lignin degradation could occur via CoA thioesters instead of the dioxygenase-dependent beta-ketoadipate pathways. The aromatic catabolism pathway with a phenylacetate CoA intermediate increased in a relative basis the most after the amendment of lignin. The phenylacetyl-CoA pathway is considered an alternate to the more classical and well-studied beta-ketoadipate pathways for aerobic aromatic catabolism (Ismail and Gescher, 2012). Unlike phenylacetyl-CoA, beta-ketoadipate has been studied in relationship to lignin degradation in multiple bacterial strains (Bugg et al., 2011), where peripheral pathways produce protocatechuate or catechol intermediates that are further degraded using ring-cleaving dioxygenases (Fuchs et al., 2011). CoA thioester pathways, in contrast, form non-aromatic ring-epoxide that may have several advantages in the marine environment (Fuchs et al., 2011). CoA thioesters are better retained by the cell which may be crucial in oligotrophic environments where more efficiency strategies are needed to survive. Also, CoA thioesters can enter both aerobic and anaerobic pathways allowing facultative anaerobic microbes to be flexible in fluctuating oxygen or low oxygen conditions. The phenylacetyl-CoA pathway has not been extensively studied in connection with lignin but may be an important mechanism in lignin transformation in the ocean.

The reads annotated as belonging to phenylacetyl-CoA pathway were linked with *Pseudomonas* and *Marinomonas* species that have previously been studied for lignin or lignocellulose degradation. The closest strain to phenylacetyl-CoA reads, *P. fluorescens* str. Pf-5, has been reported to have Dyp-type peroxidases that oxidize polymeric lignin (Rahmanpour and Bugg, 2015). The second most represented strain, *Marinomonas* sp. str. MWYL1, was originally isolated from salt marsh grass, which is a form of lignocellulosic material (Van Landschoot and De Ley, 1983). One caveat in linking phylogeny and function is that the relationship is highly dependent on quality of the database records, in this case GenBank and the source organisms on each record.

Our findings with the hemicellulose, xylan, demonstrate the utility of controls when heavily relying on cultivation-independent techniques. The lignin-adapted and xylan-adapted consortia had different functional gene abundances for key functions of aromatic and carbohydrate utilization. It is unlikely that we have a dominant xylan-degrading population within the lignin-amended consortia, which is an important distinction since lignin is often tightly complexed with xylan and cellulose even after chemical isolation. We observed much higher mineralization rates with xylan than lignin. Xylan may be preferentially utilized over lignin if both carbon sources are present, causing the community to become adapted for xylan degradation. Combining the proper controls with “omics” techniques will allow targeted studies into the biodiversity of lignin degradation in complex microbial communities.

## Conclusion

The Eastern Mediterranean seawater microbial consortium utilized lignin as a sole carbon source in a defined media. *Gammaproteobacteria* dominated the lignin-adapted community structure rather than the white-rot fungi typically studied for lignin degradation in terrestrial environments. Bacteria may be the dominant lignin-degrading species in open ocean environments. Genes corresponding to phenylacetyl-CoA aromatic catabolism pathways increased in abundance the most after lignin amendment. Phenylacetyl-CoA may be an effective strategy for marine microbes to utilize lignin in fluctuating oxygen conditions. Only one concentration of substrate was tested in this experiment. Recent evidence has shown that the low concentration rather than the structure of organic compounds in the ocean limits degradation rates (Arrieta et al., 2015). In the future, various concentrations should be tested to see if the microbial response is concentration dependent. Insight into the biodiversity of bacterial lignin degradation provides basic scientific knowledge into obtaining effective, salt-tolerant, and cheaper commercial lignin-degrading enzymes for industry and a systems-level understanding of carbon cycling in the ocean.

## Materials and Methods

### Lignin- and Xylan-Amended Enrichments of Eastern Mediterranean Seawater

The seawater for enrichment culturing was collected in Niskin bottles at 50 m below the surface of the Eastern Mediterranean Sea, off the coast of the Nile Delta. The details of the sampling cruise are described by Techtmann et al. (2015). Organosolv lignin for the enrichments was provided by Dr. Nicole Labbe of the University of Tennessee Center for Renewable Carbon. The organosolv lignin was processed at 140°C for 90 min in a solvent mixture of methyl isobutyl ketone/ethanol/water with 0.05 M H_2_SO_4_. The method is similar to the control run described by Tao et al. (2016). The organosolv lignin would have had residual quantities of xylan still attached, less than 3% of the weight on a dry basis (Tao et al., 2016).

**FIGURE 8:**
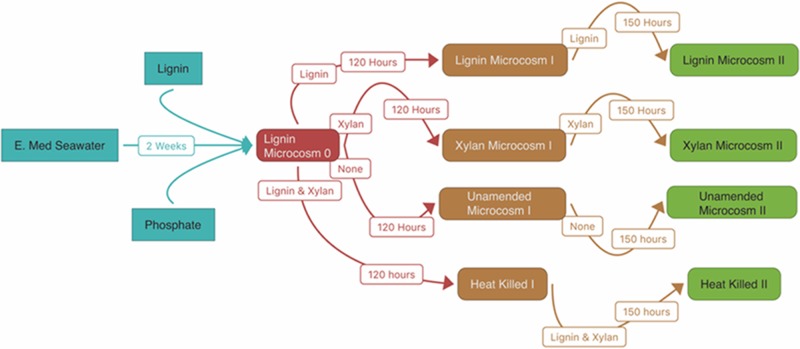
Experimental design. The arrows indicate the inoculation of each substrate-adapted consortia. The white boxes indicate the substrates amended in the microcosm and the length of the incubation.

For the first enrichment “Microcosm 0” (*n* = 1), 60 ml of seawater was enriched aerobically, in the dark, at 19°C on organosolv lignin with an extra 1.5 mM phosphate for 2 weeks. The bottle was constantly agitated at 200 rpm to allow proper aeration and mixing of the insoluble lignin. We observed carbon dioxide production within “Microcosm 0” that was four times greater than unamended controls (manuscript in preparation). After the 2-week incubation, the lignin enrichment continued by creating the two replicates of lignin enrichment, “Lignin Microcosm I,” where 1 ml of “Microcosm 0” was transferred to artificial seawater [ONR7a Medium pH 8.2 (Atlas, 2010) without bactopeptone, vitamins, and trace elements] amended with 0.05% weight to volume of organosolv lignin. In a likewise fashion, “Microcosm 0” was also transferred to xylan as a sole carbon source for the two replicates of xylan enrichment, “Xylan Microcosm I.” Enrichments were incubated for another 120 h. After the 120-h incubation, the enrichments were used to inoculate additional two replicates each of fresh 0.05% loading of substrate and fresh ONR7a media for “Lignin Microcosm II” and “Xylan Microcosm II” and incubated for another 150 h. **Figure 8** conceptually diagrams the experimental design (**Figure 8**).

### Respirometry

During the incubation, carbon dioxide production was regularly monitored every 3 h using a Micro-Oxymax respirometer (Columbus Instruments, Columbus, OH, United States). For the unamended controls of artificial seawater media only and abiotic controls of substrate-amended consortia, the cell inoculum in media was re-sterilized by autoclaving for 1 h, filtering through a 0.2-μm filter, and then inhibited from respiring using sodium azide before adding substrate.

### DNA Extraction

DNA was extracted from the lignin, xylan, and unamended enrichments. Replicates were pooled together to get enough DNA for sequencing. After the incubation, 50 ml of each consortia was harvested into pellets by high speed centrifugation at 8000 rpm. DNA was extracted from each of the pellets using a ZR-Duet^TM^ DNA/RNA MiniPrep Kit (Zymo Research Corp., Irvine, CA, United States) following the manufacturer’s instructions. DNA was cleaned of any contaminants using a OneStep^TM^ PCR Inhibitor Kit (Zymo Research Corp., Irvine, CA, United States) and then concentrated using a Genomic DNA Clean and Concentrate Kit (Zymo Research Corp., Irvine, CA, United States). DNA was prepared into two aliquots, one for 16s rRNA gene amplicon sequencing and the other for metagenomics.

### Metagenomics

The diversity of different functional genes in the enrichments was characterized using shotgun metagenomics. Shotgun metagenomics was also used to survey phylogenetic diversity more broadly to include fungi in addition to archaea and bacteria. DNA libraries were prepared using the Nextera XT DNA Library Prep Kit (Illumina, San Diego, CA, United States) following the manufacturer’s instructions. The tagmentation and amplification was verified using a Bioanalyzer High Sensitivity Chip (Agilent, Santa Clara, CA, United States). Nextera amplicon copies were quantified by real-time PCR using Illumina primers premix and Illumina DNA standards in the KK4828 KAPA library quantification kit and a BIO-RAD CFX-Connect real-time PCR thermal cycler using the manufacturer’s protocol (Kapa Biosystems, Inc., Wilmington, MA, United States). Libraries were normalized by concentration and sequenced on the Illumina MiSeq sequencing platform using a 2 × 300 basepair kit.

Unassembled but joined paired-end reads were uploaded onto MG-RAST for automated annotation (Meyer et al., 2008) using the following pipeline settings, assembled: no, duplication: yes, screening: homo sapiens, ncbi, v36, dynamic trimming: yes, minimum quality: 15, maximum low-quality base pairs: 5. Metagenome sequences are publicly available on MG-RAST. The MG-RAST accession IDs are listed in Supplementary Table S1.

SEED subsystems annotation that possess four nested categories of functions was downloaded for analysis in the R package *DESeq2* (Love et al., 2014) and visualization using *ggplot2* (Wickham, 2009). Differential abundance analysis of SEED subsystem functional categories was conducted using *DESeq2* to compare the two lignin microcosms against the two xylan microcosms. *DESeq2* is appropriate for high-throughput sequencing count data with low replication because it pools information across genes to estimate variance dispersion (Love et al., 2014).

Taxonomy of shotgun reads was derived from the “representative hit classification” of the MG-RAST annotation using the M5NR database with max *e*-value cutoff of 10^-5^, minimum identity cutoff of 60%, and minimum alignment length cutoff of 15. To find the phylogeny associated with specific functional categories, SEED subsystem annotated reads with GenBank ID were downloaded from MG-RAST. GenBank records provided source organisms for each representative hit.

### 16S rRNA Gene Amplicon Sequencing

The bacterial and archaeal community structure and diversity was characterized using 16S rRNA gene amplicon sequencing. Sequencing libraries from the DNA were prepared, purified, and quantified using the methods described in Techtmann et al. (2015). Once all libraries were multiplexed into a single sample, the sample was sequenced on an Illumina MiSeq sequencing platform using methods developed by Caporaso et al. (2012). Libraries were generated using primers 515F-806R that target the V4 region of the 16S SSU rRNA, which is appropriate for targeting bacteria and archaea only (Caporaso et al., 2011). The resulting 2 × 150 basepair Illumina reads were joined, demultiplexed, and annotated using an open OUT picking method against the Greengenes database (May 2013) using QIIME (Caporaso et al., 2010). All OTUs were chimera checked before exporting into R for additional statistics and visualization using R packages *vegan* (Dixon and Palmer, 2003), *phyloseq* (McMurdie and Holmes, 2013), and *DESeq2*. Sequences are deposited in NCBI Sequence Read Archive (SRA) under BioProject PRJNA436773 as Biosamples SAMN08631961–SAMN08631967.

The OTU table was rarefied before alpha- and beta-diversity analysis using R packages *vegan* and *phyloseq*. Taxa bar plots were created also using *phyloseq*. *DESeq2* was used on the OTU abundances without any normalization as per the R package’s instructions. The differential abundance of OTUs between the two lignin and the four “control” microcosms (two xylan-amended microcosms and two unamended microcosms) was compared using a local fitting. Indicator species of the three treatments of lignin, xylan, and the control were determined using the R package *labdsv indval* function (Roberts, 2007).

## Originality-Significance Statement

Lignin within the plant cell wall is a potential precursor for higher value products and fuels. Lignin degradation is typically associated with white-rot fungi. This is one of first microbial ecology studies investigating lignin degradation in the ocean where we found evidence supporting the notion that a diverse set of bacteria can utilize lignin as a carbon source. Metagenomic evidence suggests that lignin is degraded via the phenylacetyl-CoA pathway rather than the classical beta-ketoadipate pathway.

## Author Contributions

HW and TH conceived the study and planned the experiments, and supported the study. HW did the analysis and work, and wrote the paper with editing by TH.

## Conflict of Interest Statement

The authors declare that the research was conducted in the absence of any commercial or financial relationships that could be construed as a potential conflict of interest.
