# Supplementary material for: Enrichment of Bacteria From Eastern Mediterranean Sea Involved in Lignin Degradation via the Phenylacetyl-CoA Pathway
Source: Front Microbiol. 2018 May 9;9:922. doi: 10.3389/fmicb.2018.00922 (PMC5954211; doi:10.3389/fmicb.2018.00922)

**Supplemental Figure S1. Regression of carbon dioxide and oxygen accumulation within xylan and lignin amended microcosms.** The MicrooxyMax Respirometer monitored both carbon dioxide and oxygen accumulation in the xylan- and lignin amended microcosms. Carbon dioxide was positively correlated with oxygen utilization. A linear fit of the carbon dioxide and oxygen accumulation in moles had a high R-square of 0.90 and significant p-value less than 0.001.

**Supplemental Table S1. Metagenome sequencing results and accession numbers on MG-RAST.**

|  | MG-RAST Accession number | # Sequences | Avg %GC |
| --- | --- | --- | --- |
| Lignin Microcosm I | 4581543.3 | 608,831 | 55 |
| Lignin Microcosm II | 4581713.3 | 11,354,849 | 57 |
| Unamended Microcosm I | 4581542.3 | 5,072,350 | 56 |
| Unamended Microcosm II | 4581544.3 | 3,318,718 | 57 |
| Xylan Microcosm I | 4581712.3 | 9,134,347 | 55 |
| Xylan Microcosm II | 4581714.3 | 4,874,040 | 53 |

**Supplemental Figure S2. Number of detected functional genes in all metagenomes per SEED Subsystems category.** Categories shown corresponding the broadest level of categorization by the SEED Subsystems curated database. Individual functions are the finest level of categorization. The height of bars indicate the number of distinct individual functions detected.


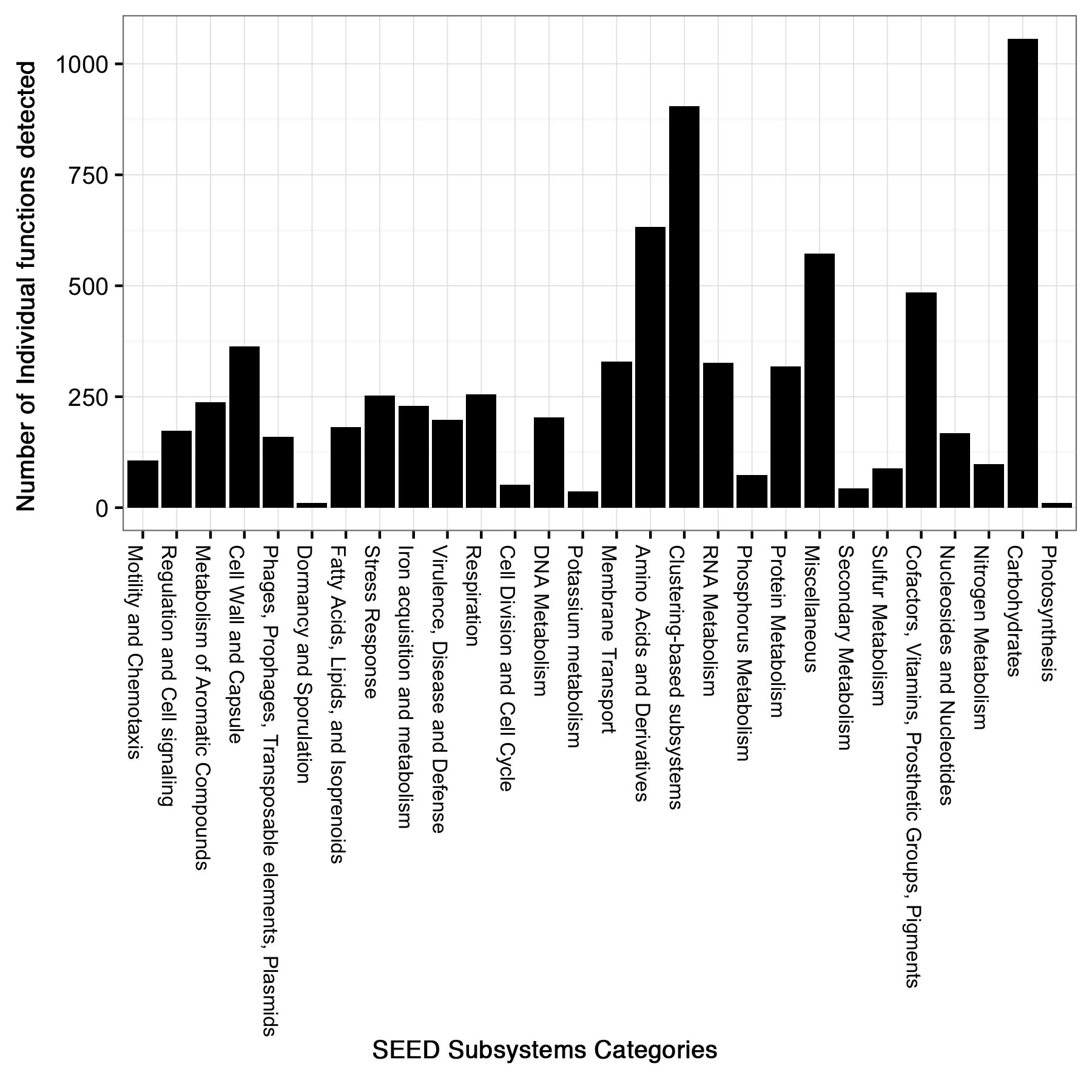


**Supplemental Figure S3. Taxa Bar plots of Archaea, Alphaproteobacteria, Gammaproteobacteria and Flavobacteria from the 16S rRNA gene amplicon sequencing.** “Others” is used to represent taxa less than 1% relative abundance.


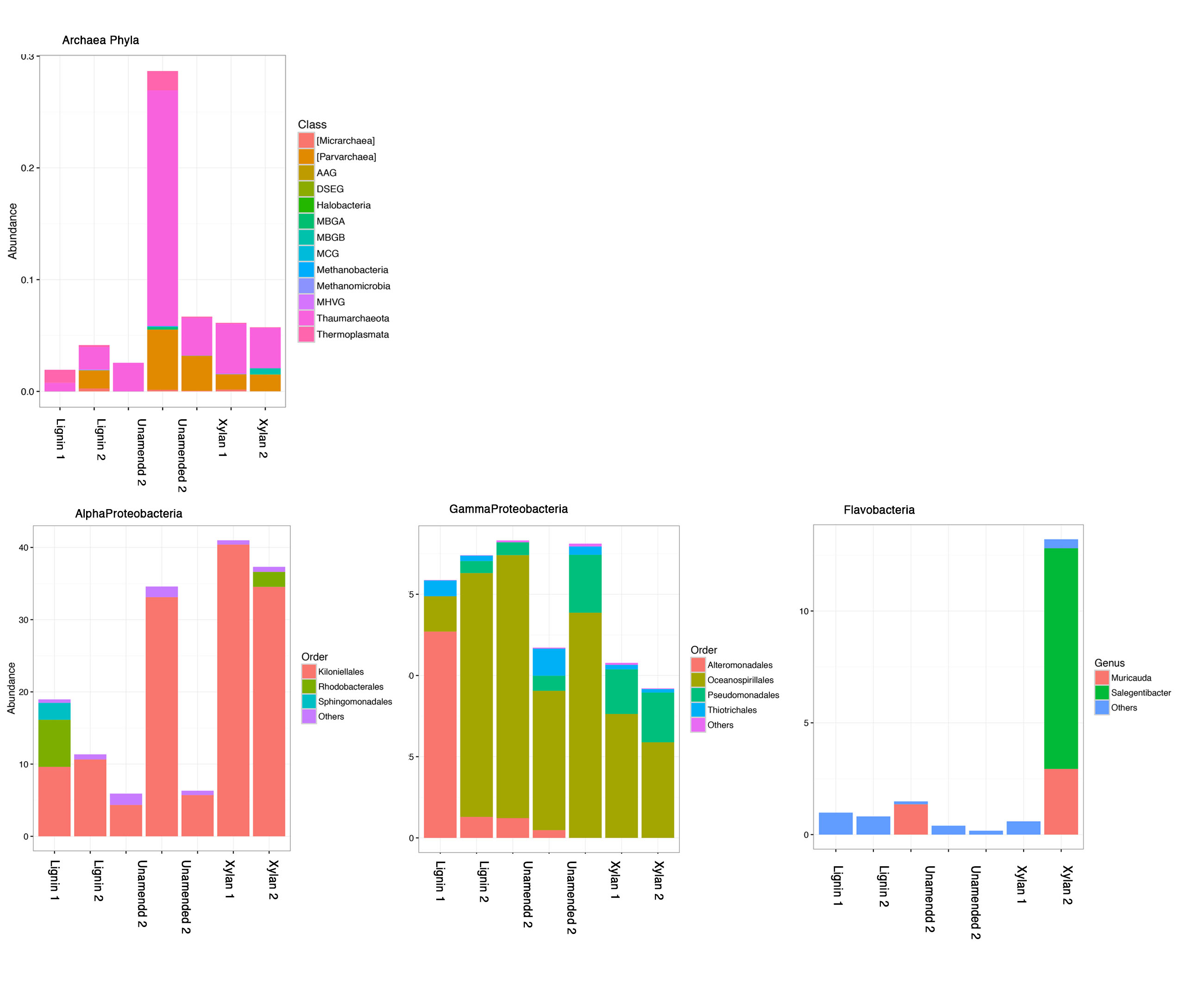


**Supplemental Table S2. The relative abundance of phyla detected in metagenomic shotgun reads.** The reads were annotated using the MG-RAST automated annotation pipeline. Table uses the “representative hit classification” from the M5NR database with a maximum e-value cutoff of 1e-5, minimum % identity cutoff of 60%, and minimum alignment length cutoff of 15.

|  | Unamended I | Unamended II | Xylan I | Xylan II | Lignin I | Lignin II | Grand Total |
| --- | --- | --- | --- | --- | --- | --- | --- |
| Acidobacteria | 0.092% | 0.075% | 0.112% | 0.158% | 0.081% | 0.100% | 0.108% |
| Actinobacteria | 1.839% | 1.308% | 0.914% | 0.857% | 1.079% | 0.999% | 1.119% |
| Apicomplexa | 0.001% | 0.001% | 0.001% | 0.001% | 0.001% | 0.001% | 0.001% |
| Aquificae | 0.025% | 0.014% | 0.020% | 0.022% | 0.018% | 0.013% | 0.018% |
| Arthropoda | 0.020% | 0.016% | 0.024% | 0.021% | 0.025% | 0.017% | 0.020% |
| Ascomycota | 0.041% | 0.028% | 0.042% | 0.036% | 0.037% | 0.030% | 0.036% |
| Bacillariophyta | 0.009% | 0.004% | 0.007% | 0.007% | 0.002% | 0.003% | 0.006% |
| Bacteroidetes | 0.783% | 0.448% | 8.116% | 23.830% | 2.083% | 4.656% | 7.280% |
| Basidiomycota | 0.007% | 0.005% | 0.006% | 0.005% | 0.006% | 0.005% | 0.006% |
| Chlamydiae | 0.011% | 0.005% | 0.012% | 0.010% | 0.006% | 0.004% | 0.008% |
| Chlorobi | 0.158% | 0.100% | 0.145% | 0.124% | 0.110% | 0.112% | 0.128% |
| Chloroflexi | 0.132% | 0.085% | 0.109% | 0.101% | 0.100% | 0.092% | 0.103% |
| Chlorophyta | 0.012% | 0.006% | 0.009% | 0.015% | 0.007% | 0.005% | 0.009% |
| Chordata | 0.073% | 0.038% | 0.065% | 0.069% | 0.050% | 0.048% | 0.058% |
| Chrysiogenetes | 0.038% | 0.028% | 0.029% | 0.023% | 0.035% | 0.029% | 0.030% |
| Cnidaria | 0.019% | 0.025% | 0.033% | 0.040% | 0.017% | 0.021% | 0.027% |
| Crenarchaeota | 0.012% | 0.012% | 0.015% | 0.012% | 0.006% | 0.012% | 0.013% |
| Cyanobacteria | 0.486% | 0.356% | 0.468% | 0.422% | 0.363% | 0.370% | 0.419% |
| Deferribacteres | 0.015% | 0.014% | 0.012% | 0.021% | 0.022% | 0.015% | 0.015% |
| Deinococcus-Thermus | 0.140% | 0.110% | 0.119% | 0.102% | 0.122% | 0.114% | 0.117% |
| Dictyoglomi | 0.001% | 0.001% | 0.001% | 0.001% | 0.002% | 0.001% | 0.001% |
| Echinodermata | 0.000% | 0.000% | 0.001% | 0.001% | 0.001% | 0.001% | 0.001% |
| Elusimicrobia | 0.003% | 0.005% | 0.005% | 0.003% | 0.009% | 0.005% | 0.004% |
| Euglenida | 0.000% | 0.000% | 0.000% | 0.000% | 0.000% | 0.000% | 0.000% |
| Euryarchaeota | 0.102% | 0.076% | 0.088% | 0.088% | 0.078% | 0.081% | 0.087% |
| Fibrobacteres | 0.001% | 0.001% | 0.001% | 0.003% | 0.001% | 0.001% | 0.001% |
| Firmicutes | 0.745% | 0.781% | 0.758% | 0.766% | 0.952% | 0.709% | 0.747% |
| Fusobacteria | 0.017% | 0.015% | 0.007% | 0.013% | 0.012% | 0.013% | 0.012% |
| Gemmatimonadetes | 0.009% | 0.011% | 0.012% | 0.014% | 0.012% | 0.012% | 0.012% |
| Glomeromycota | 0.000% | 0.000% | 0.000% | 0.000% | 0.000% | 0.000% | 0.000% |
| Hemichordata | 0.001% | 0.000% | 0.000% | 0.001% | 0.001% | 0.000% | 0.001% |
| Korarchaeota | 0.005% | 0.004% | 0.003% | 0.003% | 0.005% | 0.004% | 0.004% |
| Lentisphaerae | 0.006% | 0.007% | 0.009% | 0.011% | 0.007% | 0.005% | 0.007% |
| Microsporidia | 0.000% | 0.000% | 0.000% | 0.000% | 0.000% | 0.000% | 0.000% |
| Mollusca | 0.008% | 0.005% | 0.002% | 0.001% | 0.004% | 0.002% | 0.003% |
| Nematoda | 0.008% | 0.004% | 0.008% | 0.007% | 0.007% | 0.006% | 0.007% |
| Nitrospirae | 0.027% | 0.016% | 0.040% | 0.034% | 0.019% | 0.023% | 0.029% |
| Phaeophyceae | 0.000% | 0.000% | 0.000% | 0.001% | 0.002% | 0.001% | 0.000% |
| Placozoa | 0.001% | 0.000% | 0.002% | 0.002% | 0.001% | 0.001% | 0.001% |
| Planctomycetes | 0.132% | 0.083% | 0.127% | 0.147% | 0.096% | 0.088% | 0.113% |
| Platyhelminthes | 0.000% | 0.000% | 0.000% | 0.000% | 0.000% | 0.000% | 0.000% |
| Poribacteria | 0.001% | 0.002% | 0.000% | 0.001% | 0.003% | 0.001% | 0.001% |
| Proteobacteria | 94.341% | 95.730% | 87.993% | 72.387% | 94.022% | 91.875% | 88.836% |
| Sipuncula | 0.000% | 0.000% | 0.000% | 0.000% | 0.000% | 0.000% | 0.000% |
| Spirochaetes | 0.033% | 0.026% | 0.031% | 0.035% | 0.037% | 0.030% | 0.031% |
| Streptophyta | 0.104% | 0.060% | 0.089% | 0.077% | 0.084% | 0.057% | 0.076% |
| Synergistetes | 0.032% | 0.029% | 0.031% | 0.022% | 0.043% | 0.028% | 0.029% |
| Tenericutes | 0.003% | 0.002% | 0.001% | 0.006% | 0.005% | 0.003% | 0.003% |
| Thaumarchaeota | 0.003% | 0.001% | 0.000% | 0.001% | 0.002% | 0.003% | 0.002% |
| Thermotogae | 0.011% | 0.006% | 0.011% | 0.013% | 0.014% | 0.009% | 0.010% |
| unassigned | 0.154% | 0.127% | 0.144% | 0.117% | 0.132% | 0.128% | 0.135% |
| unclassified (derived from Archaea) | 0.003% | 0.001% | 0.004% | 0.001% | 0.002% | 0.002% | 0.003% |
| unclassified (derived from Bacteria) | 0.112% | 0.137% | 0.144% | 0.127% | 0.094% | 0.083% | 0.115% |
| unclassified (derived from Eukaryota) | 0.021% | 0.011% | 0.021% | 0.019% | 0.014% | 0.014% | 0.017% |
| unclassified (derived from Fungi) | 0.000% | 0.001% | 0.001% | 0.001% | 0.001% | 0.000% | 0.001% |
| unclassified (derived from other sequences) | 0.013% | 0.021% | 0.024% | 0.016% | 0.019% | 0.020% | 0.019% |
| unclassified (derived from unclassified sequences) | 0.053% | 0.035% | 0.042% | 0.040% | 0.040% | 0.038% | 0.041% |
| unclassified (derived from Viruses) | 0.047% | 0.072% | 0.056% | 0.044% | 0.041% | 0.047% | 0.051% |
| Verrucomicrobia | 0.089% | 0.055% | 0.080% | 0.121% | 0.070% | 0.064% | 0.079% |
| Grand Total | 100.000% | 100.000% | 100.000% | 100.000% | 100.000% | 100.000% | 100.000% |

**Supplemental Figure S4. The relative abundance of fungal taxa within the metagenomic reads.** The abundances of fungal taxa from each sample were normalized so that their sum would be 1.


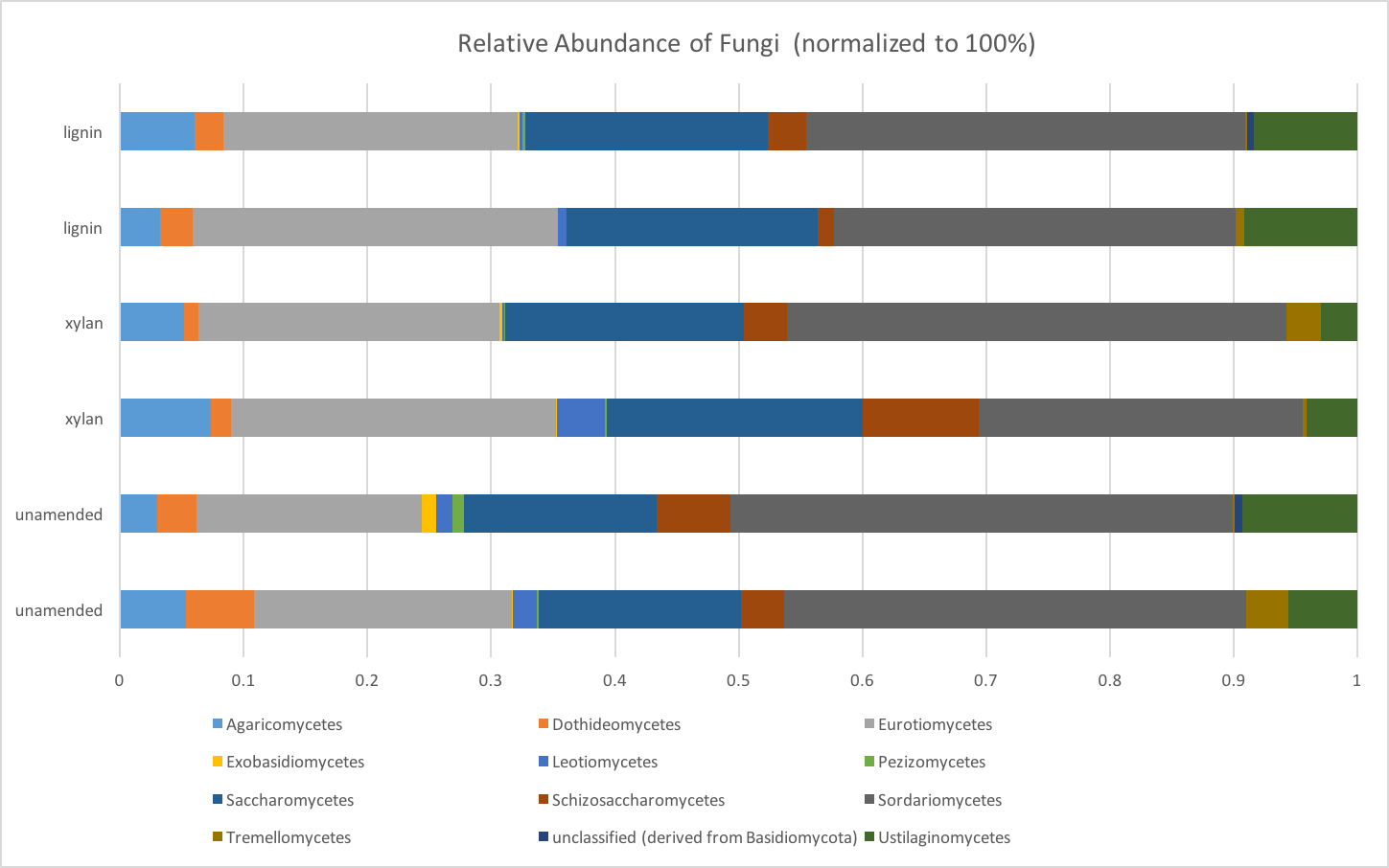

Supplement: Supplementary file 1 [file Data_Sheet_1.DOCX]
